# Promoting universal financial protection: constraints and enabling factors in scaling-up coverage with social health insurance in Nigeria

**DOI:** 10.1186/1478-4505-11-20

**Published:** 2013-06-13

**Authors:** Chima A Onoka, Obinna E Onwujekwe, Benjamin S Uzochukwu, Nkoli N Ezumah

**Affiliations:** 1 Health Policy Research Group, College of Medicine, University of Nigeria, Enugu PMB 01129, Enugu, Nigeria; 2 Department of Community Medicine, College of Medicine, University of Nigeria, Enugu PMB 01129, Enugu, Nigeria; 3 Department of Health Administration and Management, University of Nigeria, Enugu Campus 400001, Enugu state, Nigeria; 4 Department of Sociology/Anthropology, University of Nigeria, Nsukka Campus 410001, Enugu state, Nigeria

**Keywords:** Case study, Health financing, Nigeria, Social health insurance, Universal coverage

## Abstract

**Background:**

The National Health Insurance Scheme (NHIS) in Nigeria was launched in 2005 as part of efforts by the federal government to achieve universal coverage using financial risk protection mechanisms. However, only 4% of the population, and mainly federal government employees, are currently covered by health insurance and this is primarily through the Formal Sector Social Health Insurance Programme (FSSHIP) of the NHIS. This study aimed to understand why different state (sub-national) governments decided whether or not to adopt the FSSHIP for their employees.

**Methods:**

This study used a comparative case study approach. Data were collected through document reviews and 48 in-depth interviews with policy makers, programme managers, health providers, and civil servant leaders.

**Results:**

Although the programme’s benefits seemed acceptable to state policy makers and the intended beneficiaries (employees), the feasibility of employer contributions, concerns about transparency in the NHIS and the role of states in the FSSHIP, the roles of policy champions such as state governors and resistance by employees to making contributions, all influenced the decision of state governments on adoption. Overall, the power of state governments over state-level health reforms, attributed to the prevailing system of government that allows states to deliberate on certain national-level policies, enhanced by the NHIS legislation that made adoption voluntary, enabled states to adopt or not to adopt the program.

**Conclusions:**

The study demonstrates and supports observations that even when the content of a programme is generally acceptable, context, actor roles, and the wider implications of programme design on actor interests can explain decision on policy adoption. Policy implementers involved in scaling-up the NHIS programme need to consider the prevailing contextual factors, and effectively engage policy champions to overcome known challenges in order to encourage adoption by sub-national governments. Policy makers and implementers in countries scaling-up health insurance coverage should, early enough, develop strategies to overcome political challenges inherent in the path to scaling-up, to avoid delay or stunting of the process. They should also consider the potential pitfalls of reforms that first focus on civil servants, especially when the use of public funds potentially compromises coverage for other citizens.

## Background

Policy making is often characterised by much attention on the content of a health reform, but also needs to consider “the actors involved in policy reform, the processes contingent on developing and implementing change, and the context within which policy is developed” [1]. As observed by Cassells (1995), intended policies are not always in the interest of all actors, and indeed may never be [2]. More specifically, reforms involving policies for social health insurance may result in conflicts because the outcome may favour or disfavour various interest groups [3]. Similar conflicts may be observed between national and sub-national governments in environments where political power over resources and reforms is shared amongst different levels of government, typified by the federal system of government [4-6]. Such systems of government are known to allow opportunism, dynamism and self expression by sub-national governments [7]; their existence underscores the relevance of inquiry into the actor roles and influences in the policy environment that promote or constrain scaling-up of public policies.

The National Health Insurance Scheme (NHIS) in Nigeria was established by a federal government law in 1999 as a programme to help achieve universal coverage using financial risk protection mechanisms [8]. The actual implementation of the NHIS commenced in 2005 through the Formal Sector Social Health Insurance Programme (FSSHIP) that was established to cover employees of federal, state and local governments, and those of private institutions employing at least ten workers [9]. The key features of the programme are summarized in Table 1. Other programmes envisaged by the NHIS include a programme for rural dwellers, armed forces, police, and allied services, students in the tertiary institutions, voluntary contributors, and retirees.

**Table 1:** **Description of the formal sector programme of the NHIS**[8,9]

	
Key actors	• The National Health Insurance Scheme (regulators).
	• Federal, state and local governments (employers) and their employees.
	• Health Maintenance Organizations (HMOs) (paid by the NHIS to manage new enrollees, and pay capitations and reimbursements).
	• Healthcare providers (as individual doctors, pharmacists, etc., leaders of provider and practice associations, and facility owners).
Revenue collection and pooling	• The law establishing the NHIS mandates HMOs to collect contributions from employers, but in practice, NHIS does the collection. NHIS can receive additional funds including grants, donations, and also dividends and interests from investments using pooled funds.
	• The specified role of states and local governments in the FSSHIP is that they, as employers of labour, were expected to make contributions on behalf of their employees to the scheme.
	• Federal government pays the equivalent of 10% of employees’ basic salary to the NHIS, and states and local governments are expected to do the same if they adopt the FSSHIP.
	• Employee pays 5% of basic salary which is deducted at source. NHIS requires the states intending to adopt the programme to hand over the fund to them in advance and on quarterly basis.
	• NHIS pools funds at the federal level, and allocates them to HMOs to make capitation payments and fee for service reimbursements to providers on behalf of beneficiaries allocated to HMOs.
Provider	• Private and public facilities of all levels (primary, secondary, and tertiary) can provide primary care. Over 95% of providers are private.
	• Referral care is provided by secondary and tertiary facilities.
Purchasing	• HMOs disburse global capitation for primary care to providers. Referral care requires pre-approval by HMO, and provider is reimbursed on fee for service basis in relation to a fee schedule developed by the NHIS. NHIS pays administrative fees to HMOs for paying capitation and fee-for-service reimbursements.
Benefit package	• Out-patient care (including consumables), prescriptions, and diagnostic tests as contained in the National Essential Drugs List and Diagnostic Test Lists, maternity care, preventive medical and dental care, specialist consultation, in-patient care (not exceeding 15 days per year), eye examination and care, and access to locally produced prostheses.

Currently, only 4% of Nigerians (mainly federal government employees and their households) are covered by health insurance, and this is largely through the FSSHIP. Following the mandatory enlisting of federal government employees into the formal sector programme by the federal government, state governments were expected to voluntarily adopt the same programme to cover their employees and their dependents. Such an action would have greatly expanded the breath of coverage but that did not happen. However, six years after the launch of the FSSHIP, only the federal government and three out of 36 states in Nigeria – Bauchi (2008), Cross River (2007), and Enugu (2010) – had adopted the programme despite sustained advocacy by the NHIS and HMOs, suggesting the existence of constraints to adoption which need to be identified and addressed.

The paper provides information on why different state (sub-national) governments have either adopted or not the NHIS formal sector programme, and identifies strategies that encourage adoption. The paper particularly focuses on adoption of the FSSHIP at the state level rather than exploring implementation experiences, though such experiences have been examined to the extent that they influenced adoption. The information contained within the paper will provide policy makers and implementers in Nigeria and elsewhere with evidence that may enhance universal coverage reforms.

## Methods

### Study design

The study was carried out in 2011 and employed a multiple case study approach to help understand the contrasting adoption decisions made by two Nigerian states. Case studies are preferred when “how” or “why” questions are being posed, when the investigator has little control over events, and when the focus is on a contemporary phenomenon within some real-life context [10]. Multiple case studies enable comparisons between two or more case units with similar or different contexts and thereby help facilitate generalization [11].

The case units were Enugu and Ebonyi states in southeast Nigeria, which have populations of 3.26 million and 2.17 million, respectively [12]. These states were selected because they had comparable political, social, and economic characteristics but had contrasting decisions with respect to adoption. Both states have been governed by the same political party since 1999, and the citizens are predominantly civil servants, small-scale farmers, small-scale traders, Christians and of Igbo ethnic group. In 2011, the respective budgets were 36 billion naira (US$440 million) for Enugu state and 61 billion naira (US$406 million) for Ebonyi state. The FSSHIP was adopted by Enugu state government for its civil servants (though implementation was yet to commence), but not by Ebonyi state government. However, implementation of the FSSHIP commenced all over Nigeria since 2005, for federal government employees working in federal establishments across the country, and the implementation has involved health maintenance organizations (HMOs), NHIS regional officials, and public and private health care providers.

This study adopted the theoretical proposition that “the decision to either adopt or not adopt a programme is influenced by the political context, the roles played by actors who have interests in the programme, and what these actors stand to lose or gain because of the programme design”. Drawing on the Walt and Gilson framework for health policy analysis [1], the study examined the theoretical proposition by exploring the roles played by various actors and how their decision on adoption was influenced by contextual issues and the programme design.

Data were collected through document reviews and in-depth interviews (IDIs) carried out by two interviewers. Documents reviewed included state health sector policies and plans, health financing documents that were relevant to the adoption process, and laws and guidelines for NHIS programmes. Insights from the document review process, the theoretical proposition and objectives of the study, enabled generation of an initial interview guide. This was further refined during pilot studies carried out on one of each category of actors (Table 1) that were not included for the study, and during initial interviews. Interviewees included policy makers in the state government (employers), leaders of health care provider associations (providers), state level civil servant leaders (consumers/employees), and managers of the FSSHIP (regulators) and HMOs (managers) operating in these states. The interviewees either were, or should have been, involved in the adoption process at the state level.

Interviewees were asked about the way the NHIS adoption agenda was introduced in the state, the interactions between this agenda and related political, economic and health sector related activities in the state, concerns about the programme design, roles played by various stakeholders while adoption was being considered, and how such roles influenced the decision on adoption. Follow-up interviews were carried out where necessary to confirm or clarify emerging information. In total, 48 interviews were conducted in English with state level actors (26 in Enugu and 22 in Ebonyi). Ethical approval for the study was obtained from the University of Nigeria Research Ethics Committee and the World Health Organization.

### Data analysis

Interview recordings (carried out using digital voice recorders) were transcribed verbatim and the transcripts from the interviews, electronic documents and the field notes were transferred into NVivo 8 software for analysis. Data coding was carried out by two data coders, who also undertook the interviews. Initially, each coder separately undertook the coding of the transcripts. Interpretations were compared and appraised by the research team to ensure coherence. The results of the initial coding, which entailed the identification of a set of emerging themes, were compared with themes generated from the theoretical proposition to establish a coding template. Subsequently, the template was applied for further analysis with modifications made to the template as new themes arose in the analysis process. Data analysis was undertaken separately for each of the cases examined. Pattern matching was used to examine the coding results across and within the cases. Data triangulation with multiple sources of information, including documentary evidence, allowed identification of corroborating or contradicting information. Similarities and differences in views and experiences across different groups of respondents were identified and explanations were sought for key differences.

Post-study workshops in each of the states examined were used to present the preliminary findings to study participants in order to discuss and validate the analysis. The participants reached agreements on issues of interest, a number of conflicting views held by stakeholders were clarified, and consensus on the results of the analysis was reached. Particular effort was made to identify and explore views and experiences that were unusual in the context of each data set. Subsequent analysis was undertaken to reflect the discussion that occurred during the post-study workshops.

## Results

The desire of the NHIS to expand coverage of the FSSHIP in line with its operational mandate, and the interest of HMOs in attracting more members to their pools, motivated both stakeholders, acting as advocates for the FSSHIP, to independently approach both states to encourage adoption. Prompted by the NHIS and HMOs, the leadership of the Ministry of Health initiated and facilitated the agenda for Ebonyi state to adopt the programme, while in Enugu, the state governor welcomed the idea and directed a state-level team to appraise the policy and the requirements for adoption. The roles played by actors involved in appraising and deciding on adoption are presented below while the important policy context and design issues that influenced the roles of these actors are summarised in Table 2.

**Table 2:** Key policy context and design issues influencing actor roles

**Policy context**	**Ebonyi (Non-adopters of the programme)**	**Enugu (Programme adopters)**
Policy agenda of government –interest in financial risk protection	Existing public programmes for financial risk protection.	Existing public programmes for financial risk protection.
	Spends about US$100,000 monthly on free maternal and child health (MCH) services in rural areas using public and non-profit private facilities. The focus on rural areas was based on the governor’s philosophy that the share of the financial consequences of ill health was more on the more populous rural dwellers.	Funds free MCH services (state-wide using public facilities).
	Funds a vesico-vaginal fistula programme.	No medical allowance for civil servants though previously requested by them.
	Medical allowance (10% of basic salary paid to civil servants monthly to help defray health care expenditure).	
Role of states in the FSSHIP	States as federating units are allowed to deliberate on health policies even when approved by the federal government. Concerns were raised that the scheme was a federal government programme which meant states had to transfer funds to the federal level for a scheme which was established by federal law that did not specify a role for states apart from their broad inclusion as employers of labour.	Concerned about absence of role for states and initially considered setting up a state level health insurance scheme.
	Civil servants and policy makers had previous negative experiences with a contributory federal led programme (national housing fund): they made contributions, were yet to enjoy the benefits, and could not retrieve their funds.	The choice of the FSSHIP was made to take advantage of existing institutional structure and technical capacity for managing insurance considered lacking in state but available in the NHIS.
Accountability systems for FSSHIP	Concerns expressed by policy makers that the NHIS had not presented any audited report to the state or the general public since its inception which created the feeling of distrust towards the scheme. This view was also corroborated by HMOs.	Accountability issues were not raised during the adoption process.
**Design issues**		
Employer contribution	Policy makers considered the level of employer contribution (10%) acceptable and economically feasible as long as the already paid medical allowance would be reallocated to the programme.	Government was willing to make contribution.
Employee contribution	Civil servants considered wages too low to allow deductions even though the rate of 5% was considered reasonable.	Civil servants considered capitation rate reasonable.
	
		Wanted payment deferred and to allow them time (at least one year of benefiting from the programme) to be convinced about actor commitment to implementation.
		Also felt they would not be asked to contribute in the long run because NHIS had allowed federal employees not to pay since inception of the programme in 2005, which suggested that employer contribution was sufficient for running the FSSHIP.
Capitation rates and drug list	Generally considered inadequate by providers.	Considered inadequate by providers given that the failure of the NHIS to revise the rates within the first 6 years of implementation.
	Most providers report frequent conflicts with dissatisfied federal employees that they provide services to for two main reasons: low capitation and an unrevised schedule of drugs (since 2005) that meant patients had to buy unlisted drugs out of pocket.	Concerns about drug schedule which had not been revised.
Benefit package	Considered sufficient enough to address most needs of civil servants and their households.	Same as for Ebonyi state.

### Case one: Ebonyi state (non-adopters of the programme)

The request made by the NHIS for the state to ‘fold into the scheme’ as an employer of labour was interpreted by policy makers to mean handing over state funds to a federal government agency. Key policy makers in the state government considered both the absence of a governance role for states in the running of FSSHIP and a perceived lack of transparency in the NHIS, which makes it *“difficult for us to do a blind transfer of state money to them (NHIS)”* (Policy Maker), as major impediments to adoption. The contentious issue which was yet to be resolved was the level of government that would hold the contributions.

*“States have been arguing to have custody of the fund, but the federal says ‘No; this is our parastatal’. The misunderstanding has made people to lose interest”* (Policy Maker, Ebonyi).

In addition, policy makers, like civil servants, reported the absence of positive reports from neighbours and friends about the FSSHIP (which they considered more valuable than media adverts employed by the NHIS), and also the potential negative implications of not having control over their money (which was shaped by previous experience with another federal contributory scheme), as impediments to adoption. To the policy makers, *“The natural or real local testimonies from federal workers living in the state”* (Policy maker) were not forthcoming, but such evidence would have made employees allow deductions from their salaries.

Despite reluctance towards adoption, the government, through the Ministry of Health, engaged with civil servants to appraise their interest in adoption. However, this was on the premise that civil servants would have to part with the medical allowance already being paid to them, which the government would reallocate as employer’s contribution for the FSSHIP. This meant that civil servants would part with their medical allowance (10% of the basic salary), apart from the employee contribution of 5% of their basic salary. Both policy makers and civil servants considered this impracticable with the latter deciding not to support adoption because they were unconvinced that the access they would gain to the programme benefits would be sustained in the long-run, given the risk that the scheme may collapse *“like the National Housing Fund which failed”* (Civil servants’ leader). They therefore opted to retain control over their existing medical allowances rather than have it converted to contributions for the FSSHIP. This position eased local pressure on the government for adoption.

*“There is nothing pushing us to adopt it because if the workers were interested, they could have asked like they do for other things. They seem to be comfortable with the medical allowance already approved for them”* (Policy maker).

As a strategy to overcome the prevailing challenge, the NHIS, HMOs, and civil servants suggested that the government should release fresh funds to make-up the employer contributions, but the suggestion was rejected by the government who insisted that there was a moral responsibility for the government to spend remaining funds on other citizens since civil servants were already receiving medical allowance. This caused HMOs and NHIS programme managers to deem the governor (seen as the prime determinant of the adoption decision) and his government uninterested in adoption.

*“I can tell you honestly, having interacted with many of them [policy makers and NHIS officials] during our [advocacy] visits, the main person there is the governor. If the governor should wake up today to say, ‘I’m doing this thing [adopting the programme], let me just take the risk’, he will do it. The other policy makers and the NHIS have their limits. They will only send proposals, make recommendations; but it is left with the big man to adopt it. If he says the government does not have money to do it, there is nothing you can do”.* (HMO manager).

Requests by the NHIS and HMOs for meetings to better inform the state executive council about the benefits of FSSHIP were ignored by key policy makers who argued that the government’s concerns (which were not about benefits) were known, and no request had been made to discuss strategies to address the concerns. Moreover, policy makers felt that rather than persist in asking the state government to make new employer contributions to enable adoption, the NHIS and HMOs should have taken advantage of the governor’s interest in the welfare of rural dwellers and suggested ways of developing successful health insurance products for this group. They believed that such a strategy could have been used to prompt positive testimonies that would have encouraged civil servants to use their medical allowance for the FSSHIP. According to a policy maker, *“If federal government sends a proposal, we can then say, let’s do it our own way; another state’s proposal may not make much sense to us”.* The NHIS, focused on the FSSHIP at the time and unable to force the state to adopt the programme, shifted attention to other states. As observed by a NHIS manager, *“If it were an activity that only the federal government can legislate, a state government will have no choice than to accept it; but we cannot impose it on them, since they [states] have the authority to accept or to refuse it based on their own legislation. This is why we are trying to change the law to make it mandatory”*. On their part, HMOs were reluctant to invest significantly in the state because of the alleged policy makers’ disinterest in the scheme and uncertainties about the process of HMO selection. Both HMOs and the NHIS also did not engage with civil servants to discuss options for addressing the problem of employee contributions.

A general state of apathy also existed amongst health provider associations (physicians and pharmacists) though the leaders insisted that they could have put pressure on the government if adoption had been in their interest. These interests, however, differed. The position of the physicians’ union was attributed to frequent complaints by members providing health services under the FSSHIP to local federal employees about the inadequacy of capitation, which had not been revised for the six years since implementation. In addition, union members reported that patients often expressed dissatisfaction with services and attributed this to an out-dated drug list that left patients making drug purchases out-of-pocket, while providing HMOs with additional profits. Members believed that these failures negatively affected their reputation and discouraged members from supporting adoption.

Pharmacists believed that the practice of global capitation coupled with the low capitation, made facility owners (doctors) use only facility-owned drugs rather than issuing prescriptions for patients to obtain drugs from registered pharmacies. Their observation that facility owners employed and controlled their own staff and drug-management systems foreclosed the expected independent-assessment of prescriptions, and made pharmacists irrelevant actors in the FSSHIP.

*“Doctors use their clinic, and diagnose, prescribe and dispense at the same time. They do not send patients to private pharmacies. The proper thing should be that you prescribe, you move, then the next person handles his own aspect, everybody will be involved and can check the other person in the interest of the patient. Let us not do it as if we are trying to kill the pharmacy profession”* (Leader, pharmacists’ association).

While HMOs denied making excess profits from the capitation problem, a HMO manager noted that low capitation was “a burning issue” whenever it was raised among providers. However, the NHIS believed that low capitation, although an important issue to address, was not the main issue; rather, they felt that most providers still did not understand the cross-subsidising nature of insurance.

### Case two: Enugu state (programme adopters)

The successful adoption of the programme in Enugu was attributed by interviewees to the initiation of the agenda and leadership provided by the state governor, who showed sustained interest in adoption, and set up and monitored a technical committee that considered FSSHIP design and the feasibility of adoption. Having appraised various issues of interest (Table 2), the state government initially considered modifying the design such that a state-level health insurance scheme would operate the programme, and where that did not work out, to directly contract with HMOs to run the programme. The aim was to retain state government’s control over its funds. Following an acknowledgement of the paucity of local capacity for managing the scheme, the government adopted the FSSHIP, after making an unsuccessful attempt to secure a reduction in the employer contribution rate. This action was informed by the perceived lack of effort exhibited by the NHIS towards collection of contributions from federal employees and the existence of cheaper health insurance products offered to informal sector workers by some HMOs, both of which suggested that employer and employee contribution rates could be reduced.

While the state contemplated the decision, the NHIS national leadership made a strong advocacy visit to the governor, agreeing to overlook employee contributions in the interim period as demanded by civil servants. Its regional office subsequently provided a road map for adoption, participated in state technical meetings, and supported government’s intention to pass a law that would sustain adoption. The willingness of the governor to release funds for employer contributions roused the interest of policy advocates. Consequently, HMOs supported adoption by financing a workshop to help allay worker fears, providing guidance to policy makers during their negotiations with the NHIS, and by encouraging the passage of the law to sustain adoption and to avoid termination of the programme if a change of government occurred, as had happened in another state. With previous fears amongst policy makers minimised, the law establishing the scheme passed by the state house of assembly ended up being a two-page document that essentially declared adoption in line with existing NHIS guidelines.

The decision of civil servants to support adoption resulted from efforts they made to understand the concept of health insurance and the FSSHIP design, with the help of HMOs, and their successful negotiation of a favourable position (not to make employee contributions in the short term) during engagements with policy makers and legislators. They declared informed (conditional) endorsement for adoption through a communiqué sent to the government’s technical committee.

On their part, leaders of doctors and pharmacists in the state raised similar concerns as their counterparts in Ebonyi state with respect to capitation, and were opposed to adoption. Doctors considered the capitation amount of 550 naira (US$3.6) too small to allow them *“Pay a pharmacy when someone buys drugs there*” (Medical union leader). Given the small amount, it was also considered inappropriate to lump various provider services within it, knowing that “*Nobody wants to release the one he has*” (Medical union leader). Providers however observed that though insufficient capitation reportedly stirred dissatisfaction amongst existing FSSHIP beneficiaries, user complaints appeared minimal because enrollees had no financial commitment to the programme. Nonetheless, providers were faced with the added duty explaining inadequacies in the system, and this situation further made them unwilling to promote the programme to patients and government. However, unlike other actors, professional health provider associations were not involved in the adoption process, were not included in the technical committee, and were unaware of the public hearing to consider adoption. This meant that their concerns, which pitched them against adoption, were not taken into account. Policy makers considered this an oversight rather than a deliberate act, but believed the outcome of adoption should be acceptable to providers as the primary economic beneficiaries of adoption.

## Discussion

These case studies show that adoption occurred or not, not necessarily due to the content of the programme design, but due to the political and economic interests of actors involved in the scheme and the roles played or not by those actors given the policy context and programme design. The findings support the observation that health reform is a highly political process involving many actors within a state or society (including policy makers, health providers and consumers) with interests that may be affected by a proposed policy change [1,3,13-16]. Both cases were similar with respect to existence of financial risk protection measures covering some citizens of the state (pregnant women and children), positive perceptions about the benefit package, an absence of local pressure on policy makers by civil servants and providers, employees’ views about contributions, and provider concerns about capitation and the drug schedule. They however differed significantly in the interests of actors and roles they played. Three key factors that influenced adoption are presented below along with recommendations on how to improve on them to encourage adoption especially in settings where political power over resources and reforms is shared amongst different levels of government.

### Adoption is influenced by contextual factors such as the way political power is shared and used by actors at various levels of government

Policy advocates have encouraged the adoption of the FSP by states within the context of ‘power sharing’ between national and sub-national levels for public policies. The power of state governments over state-level health reforms, attributed to the prevailing federal system of government that allows states (federating units) to deliberate on certain federal government policies such as the NHIS, was exercised in the policy appraisal process and shaped its outcome. This was further enabled by the NHIS legislation that made adoption voluntary, and the non-inclusive nature of the NHIS Act, which overlooked states in its governance structure, thereby giving them no influence over use of their own funds. Poor accountability posturing at the NHIS, given previous negative experiences of state level actors only further encouraged sub-national resistance. Scaling up processes require sub-national support and leadership [17]. Garnering support would even be more expedient in setting as the one studied here, where states wield power over reforms. However, the top-down scaling-up approach used for the FSSHIP created opportunities for states to either consider modification of the design to suite local interests or to sideline deliberations on adoption, thereby threatening the intentions of the policy advocates.

### Though design issues may be acceptable, adoption is affected by considerations about the feasibility of implementing them

In Ebonyi state adoption was considered unfeasible because additional funds would be allocated to civil servants’ welfare, potentially at the expense of the welfare of other citizens who were of political interest to the government. The defining opportunity of using medical allowance (deployed in Enugu state that previously had no such commitment to workers) was no longer available to the government, but the government had already made that commitment and civil servants were happy with it. From the governments’ point of view, adoption is only feasible if resources are allocated fairly towards the welfare of various categories of citizens. Allocating new funds to civil servants’ welfare was not appealing as it could threaten this equilibrium and limit the government’s intentions for other citizens. Political feasibility, coupled with earlier noted concerns about transparency and programme effectiveness, may underlie the unwillingness to adopt the FSSHIP by 33 of the 36 states in Nigeria.

Apart from employee contribution, it also appears difficult to commence collection of employee contributions both at the national and now the state level. While, social health insurance (SHI) typically requires wage earners to make contributions [18-21], there are concerns that SHI implementation strategies that initially focus on civil servants (who are easier to identify and cover) may impact negatively on those not covered, and consequently, derail efforts towards universal coverage [22]. The growing unwillingness by civil servants to make contributions (at both federal and state levels) implies underfunding of the FSSHIP, a perpetuation of the use of public funds to provide ‘free’ services to civil servants, who make no contribution towards social health insurance, and a further erosion of the possibility of using public spending to cover other citizens.

### Adoption at sub-national levels is influenced by the position and influence of key actors at this level

Just as the interest and position assumed by national leaders acting as reform drivers affect the speed of reforms [3], this study shows that at sub-national levels, similar situations occur for policy adoption. Apart from the political context, perceived and actual problems in the NHIS including its accountability shortcomings, alleged shortage of local evidence of impact and implementation challenges amongst providers, were observed by and influenced the position of all local actors. Consequently, there were critical differences in the roles of policy champions (i.e., key policy makers, such as the governor, that largely determine the direction the government takes on a specified agenda). The state governor in Enugu, opting to adopt the programme, served as a rallying point for the process, while the government’s decision to make employer contributions became a critical enabling factor for adoption. Conversely, the key leaders in Ebonyi were opposed to adoption, and restricted engagements with the policy advocates of the FSSHIP, even though they allowed the Ministry of Health, acting as the prime driver to engage with other actors. Nonetheless, dismissing the state government as being disinterested in the FSSHIP adoption, when the NHIS and HMOs could have taken advantage of the government’s financial commitment towards the welfare of disadvantaged groups to develop and test products for other citizens such as rural dwellers appears to be a missed opportunity. Such policy options that address political interests of local policy makers need not be ignored by policy advocates, as the success in creating desired ‘local testimony’ through them, may additionally temper the resistance of civil servants towards allowing salary deductions, and enhance the chance of adoption of the FSSHIP in the long run.

The observation that civil servants would take a relatively neutral stance when not asked to pay to become members of the scheme, but resist adoption when asked to pay, also shaped the FSSHIP scale-up. While the reason for resistance or disinterest amongst providers was mainly financial, it appeared (for civil servants) to be due to ‘distrust’ – they were interested only to the extent that the income they were already certain about would not be ploughed into a programme which could collapse after a while. Such programme failure would jeopardize their access to the benefits of the scheme and also leave them without a guarantee that they would recover their money. The complementary roles played by the NHIS and HMOs facilitated opportunities for engagement with the policy makers to clarify concerns that otherwise would hinder adoption in one state. The engagements present in Enugu, which facilitated temporary waiver of employee contributions and legislation to help ensure programme sustainability, were lacking in Ebonyi and can be considered an important factor in creation of trust in the scheme amongst civil servants.

Finally, the findings that health care provider disinterest seemed to impede the adoption process in one state and the exclusion of providers in the policy discussion enabled adoption in the other state reflects their importance as local actors in the adoption process. The global capitation system used for the programme pitched independent pharmacists against doctors as it has excluded them from being primary financial beneficiaries even though they were originally accredited as providers. Such conflicts are at the detriment of adoption. Studies have demonstrated the importance of health providers as ‘street level bureaucrats’ whose engagement with patients and the policy making process can influence policy decisions and implementation [23-26]. Ignoring provider concerns during adoption may only shift policy resistance to the implementation period.

While the use of two cases (states) with contrasting decisions on adoption of FSSHIP serves as a major strength of this study, the use of only two cases may have limited the scope for exploring factors affecting states’ decisions on adoption. The position of the researchers as outsiders in the adoption process may have also limited the information accessible to them, but also enhanced the willingness of the different actors to share information with them. The fact that the actors interviewed already knew about FSSHIP and may have provided services for federal employees residing in the state significantly shaped their views about the programme. Nonetheless, the findings of this study highlight the value of using multiple rather than single case studies, in policy analysis and the value of qualitative methods in understanding health policy reforms [1,10]. Overall, this study suggests that the NHIS first needs to resolve problems with existing FSSHIPs in order to earn the trust of stakeholders because the perceived and actual problems are observed by and influence all state level actors. For instance, the NHIS council, in line with the legal provisions establishing it, should commence the annual publication of reports on its activities and its audited accounts. Such information should be made available to all states, which should have well-defined governance roles, whether or not they have adopted the programme, since all states are being courted to adopt. Given the political context, it is uncertain if an NHIS Act that mandates states to adopt its programme will work out in practice or, alternatively, whether efforts at scaling-up would yield better results if states had authority over some aspects of the programme including resource control. Capitation rates and the schedule of drugs need to be revised to encourage provider support for adoption. The reason for the inability of the NHIS to collect employee contributions also needs to be identified, understood, and addressed in order to incentivize civil servants to release employee contributions. Not doing so may jeopardize the future of the programme. A focused exploration of the implementation process of the FSSHIP will also be necessary to better inform efforts at addressing concerns impeding adoption.

## Conclusions

This study demonstrates and supports observations that even though the content of a programme is generally acceptable, a favourable context, actors’ roles, and wider implications of the design on actors’ interests explain decisions concerning adoption. Policy implementers involved in scaling-up the programme should develop strategies to address context-related challenges of individual states, such as the inability to reallocate funds into the programme, in order to assist states overcome hindrances to adoption. Policy implementers also need to be aware that policy adoption can be influenced by perceptions about the effectiveness of an existing, corresponding programme and such perceptions may hinder adoption out-rightly or shape the outcome of the adoption process.

Policy makers and implementers in countries scaling-up health insurance coverage need to carefully consider potential pitfalls of employing universal coverage strategies that first focus on civil servants, especially when providing coverage to this group using public funds may potentially compromise the availability of financial risk protection measures to other citizens. In addition, it should be noted that decisions to allow a period of time for civil servants to ‘test’ the benefits of a programme before they start making contributions may be difficult to reverse and may become a reference point that generates resistance amongst prospective contributors. There is also a need to recognize the importance and interests of policy champions, such as state governors, in pushing health reforms to enhance the chances of policy adoption. Finally, health care providers need to be incorporated into shaping adoption of policies or programmes to ensure desired outcomes are achieved without resistance from this group.

## Abbreviations

FSSHIP: Formal sector social health insurance programme; HMOs: Health maintenance organizations; IDIs: In-depth interviews; NHIS: National health insurance scheme; SHI: Social health insurance

## Competing interests

The authors declared that they have no competing interest.

## Authors’ contributions

CO conceived the study. All authors developed the study protocol and participated in its execution. CO wrote the first draft while all the authors reviewed the manuscript and approved the final document.

## Authors’ information

All authors are researchers at the Health Policy Research Group, University of Nigeria.

## References

[B1] Walt G Gilson L Reforming the health sector in developing countries: the central role of policy analysis Health Policy Plan 1994 9 4 353 370 10.1093/heapol/9.4.353 10139469

[B2] Cassells A Health Sector reform: key issues in less developed countries. Discussion paper number 1 1995 Geneva: World Health Organization, Forum on Health Sector Reform

[B3] Thomas S Gilson L Actor management in the development of health financing reform: health insurance in South Africa, 1994-1999 Health Policy Plan 2004 19 5 279 291 10.1093/heapol/czh033 15310663

[B4] The American Heritage The American Heritage Dictionary of the English Language 2000 4 Boston, MA: Houghton Mifflin Company

[B5] Elaigwu JI Nigerian federalism under civilian and military regimes Publius J Federalism 1988 18 1 173 188

[B6] Lee PR Estes CL New federalism and health policy Ann Amer Acad Political Social Sci 1983 468 88 102 10.1177/0002716283468001006 11631432

[B7] Nathan RP Federalism and health policy Health Aff (Millwood) 2005 24 6 1458 1466 10.1377/hlthaff.24.6.1458 16284017

[B8] National Health Insurance Scheme Decree No 35 of 1999, Laws of the Federation of Nigeria [http://www.nigeria-law.org/National%20Health%20Insurance%20Scheme%20Decree.htm] [Accessed 10^th^ December, 2011]

[B9] NHIS Operational Guidelines 2005 Abuja: National Health Insurance Scheme

[B10] Yin RK Case Study Research: Design and Methods (Applied Social Research Methods Vol 5) 2009 4 Thousand Oaks, CA: SAGE Publications Inc

[B11] Walt G Shiffman J Schneider H Murray SF Brugha R Gilson L ‘Doing’ health policy analysis: methodological and conceptual reflections and challenges Health Policy Plan 2008 23 5 308 317 10.1093/heapol/czn024 18701552 PMC2515406

[B12] State Population [http://www.population.gov.ng/index.php/state-population] [Accessed 12th December, 2012].

[B13] Agyepong IA Adjei S Public social policy development and implementation: a case study of the Ghana national health insurance scheme Health Policy Plan 2008 23 2 150 160 18245803 10.1093/heapol/czn002

[B14] Gilson L Doherty J Lake S McIntyre D Mwikisa C Thomas S The SAZA study: implementing health financing reform in South Africa and Zambia Health Policy Plan 2003 18 1 31 46 10.1093/heapol/18.1.31 12582106

[B15] Gonzalez-Rossetti A Bossert TJ Enhancing the Political Feasibility of Health Reform: A Comparative Analysis of Chile, Colombia, and Mexico 2000 Cambridge, MA: USAID funded Data for Decision Making (DDM) Project

[B16] Grindle MS Thomas JW Public choices and policy change: the political economy of reform in developing countries 1991 Baltimore: John Hopkins University Press

[B17] Schneider H Coetzee D Van Rensburg D Gilson L Differences in antiretroviral scale up in three South African provinces: the role of implementation management BMC Health Serv Res 2010 10 Suppl 1 S4 10.1186/1472-6963-10-S1-S4 20594370 PMC2895748

[B18] Carrin G Social health insurance in developing countries: A continuing challenge Int Soc Secur Rev 2002 55 57 69

[B19] Carrin G James C Reaching Universal Coverage Via Social Health Insurance: Key Design Features In The Transition Period. Discussion paper number 2 2004 Geneva: World Health Organization

[B20] Normand C Weber A Social Health Insurance: A Guidebook for Planning 2009 2 Geneva: WHO

[B21] Mossialos E Dixon A Mossialos E, Dixon A, Figueras J, Kutzin J Funding health care: an introduction Funding Health Care: Options for Europe 2002 Buckingham, PA: Open University Press 1 30

[B22] WHO The World Health Report 2010: Health Systems Financing, The Path to Universal Coverage 2010 Geneva: World Health Organization 10.2471/BLT.10.078741 PMC2878164 20539847

[B23] Kamuzora P Gilson L Factors influencing implementation of the community health fund in Tanzania Health Policy Plan 2007 22 2 95 102 10.1093/heapol/czm001 17299023

[B24] Walker L Gilson L ‘We are bitter but we are satisfied’: nurses as street-level bureaucrats in South Africa Soc Sci Med 2004 59 6 1251 10.1016/j.socscimed.2003.12.020 15210096

[B25] Gilson L Kalyalya D Kuchler F Lake S Oranga H Ouendo M Strategies for promoting equity: experience with community financing in three African countries Health Policy 2001 58 1 37 67 10.1016/S0168-8510(01)00153-1 11518601

[B26] Lipsky M Street-level Bureaucracy: Dilemmas of the Individual in Public Services 2010 New York: Russell Sage Foundation

